# Elucidating the Role of the T Cell Receptor Repertoire in Myelodysplastic Neoplasms and Acute Myeloid Leukemia

**DOI:** 10.3390/diseases13010019

**Published:** 2025-01-17

**Authors:** Georgios Petros Barakos, Vasileios Georgoulis, Epameinondas Koumpis, Eleftheria Hatzimichael

**Affiliations:** 1 First Department of Internal Medicine, General Hospital of Piraeus “Tzaneio”, 18536 Piraeus, Greece; bargeo46@gmail.com; 2 Department of Haematology, Faculty of Medicine, School of Health Sciences, University of Ioannina, 45500 Ioannina, Greece; vasileios.georgoulis@gmail.com (V.G.); an.koumpis@uoi.gr (E.K.)

**Keywords:** myelodysplastic syndromes, acute myeloid leukemia, T cell receptor, TCR repertoire, immunotherapy, hematopoietic stem cell transplantation

## Abstract

T cells, as integral components of the adaptive immune system, recognize diverse antigens through unique T cell receptors (TCRs). To achieve this, during T cell maturation, the thymus generates a wide repertoire of TCRs. This is essential for understanding cancer evolution, progression, and the efficacy of immunotherapies. Myelodysplastic neoplasms (MDS) and acute myeloid leukemia (AML) are hematological neoplasms that are characterized by immune evasion mechanisms, with immunotherapy giving only modest results thus far. Our review of TCR repertoire dynamics in these diseases reveals distinct patterns: MDS patients show increased TCR clonality with disease progression, while AML exhibits varied TCR signatures depending on disease stage and treatment response. Understanding these patterns has important clinical implications, as TCR repertoire metrics may serve as potential biomarkers for disease progression and treatment response, particularly in the context of immunotherapy and stem cell transplantation. These insights could guide patient stratification and treatment selection, ultimately improving therapeutic outcomes in MDS and AML.

## 1. Introduction

The ability of T lymphocytes (T cells) to generate an effective immune response against various tumor T cell antigens is dependent on the existence of a diverse T cell receptor (TCR) repertoire. This diverse T cell pool is essential for preventing cancer development and progression and for immunotherapeutics like checkpoint blockade [1]. Analyzing the TCR repertoire provides insights into the tumor microenvironment and immune responses during cancer progression. It also aids vaccine development through neoantigen identification [1]. Myelodysplastic neoplasms (MDS) and acute myeloid leukemia (AML) are hematologic malignancies associated with immune response failure and evasion, with T cell behavior playing a major role [2].

MDS are a group of myeloid malignancies characterized by dysplasia in bone marrow cells, cytopenias in the peripheral blood, and a risk of transformation to AML [3]. The immune milieu in MDS exhibits distinct dysregulation patterns in low-risk and high-risk diseases, both within a proinflammatory environment [2]. In low-risk MDS, the T cell compartment shows increased CD8+ T cells with autoreactive potential [2], with early spectroscopic studies showing skewing and oligoclonality [4]. On the contrary, higher-risk diseases are associated with CD8+ T cell impairment and regulatory T cell (Tregs) expansion, resulting in suppression of the antitumor response [2]. Allogeneic hematopoietic stem cell transplantation (allo-HSCT) is the only approved and applied immunotherapy for MDS. Several other immunotherapies that harness the power of the T cell immune response (immune checkpoint inhibitors (ICIs), adoptive cell therapy, and vaccines) show mixed results in early-phase clinical trials, reviewed in [5].

AML is a highly aggressive and heterogeneous malignancy of myeloid stem cells that frequently leads to fatal bone marrow failure [6]. The immune landscape of AML shares some similarities with high-risk MDS, marked by reduced CD8+ T cell functionality and effective immune evasion [2]. Similarly to MDS, besides allo-HSCT, the field of immunotherapy in AML is under active clinical investigation, with many issues regarding efficacy stemming from the immune evasive properties of the AML clone and its microenvironment [7].

However, little is known about the dynamic change of the TCR repertoire of patients with MDS or AML; its pathophysiologic explanation; and its potential diagnostic, prognostic, or therapeutic implications. In this review, we will discuss the research regarding the role of the TCR repertoire in MDS and AML disease progression and its importance in immunotherapy, after a brief outline of the TCR biology.

## 2. General Aspects of TCR Biology

**Figure 1:**
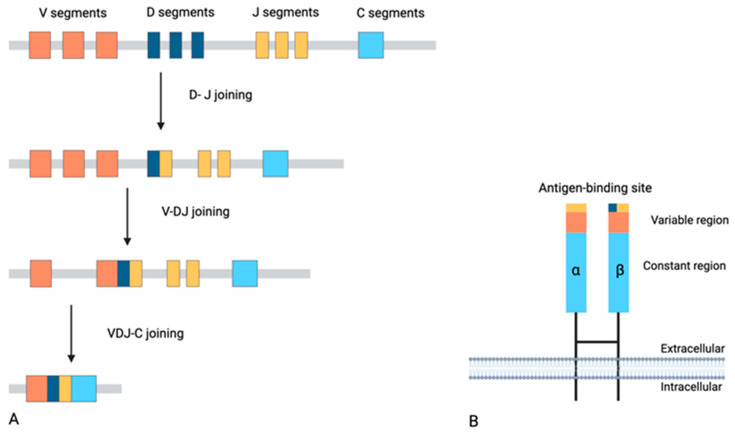
Schematic diagram illustrating the V(D)J recombination process of the β chain (**A**) and an assembled TCRαβ (**Β**). Constant regions and gene segments (C) are represented in light blue. Variable (V), diversity (D), and joining (J) are shown in orange, dark blue, and yellow, respectively. Created in BioRender. Barakos, G. https://BioRender.com/d60w564 (accessed on 3 December 2024).

T cells, along with B cells, are the most important components of adaptive immunity, spearheading cellular immunity as well as aiding in humoral immunity and immune cell recruitment through cytokine production [8]. To elicit an effective immune response against diverse antigens [from infectious agents to tumor-associated antigens (TAAs) and cancer neo-antigens], a manifold of different and unique TCRs—the TCR repertoire—is crucial [9,10]. In the course of positive and negative selection of thymocytes in the thymic cortex and medulla, respectively, TCRs capable of antigen recognition are produced via TCR-gene rearrangements [11]. Each functional TCR is a heterodimer on the surface of T cells, consisting of two paired protein chains, with the majority being comprised of paired alpha (α) and beta (β) chains [12] (Figure 1). These TCRαβ recognize antigens after presentation from major histocompatibility complex (MHC) class proteins [12]. TCRγδ-positive T cells (1–5% of all T cells) engage in innate immune responses in an MHC-unrestricted manner, with their ligands still being largely undefined [13].

Regarding structure, all TCR chains comprise a variable region (located in the N-terminus) and a constant region (located in the C-terminus) [12]. The variable region is constructed from variable (V), diversity (D), and joining (J) gene segments through the V(D)J recombination process. α (T cell receptor alpha-TRA) and γ (T cell receptor gamma-TRG) chains contain V and J gene segments, whereas β (T cell receptor beta-TRB) and δ (T cell receptor delta-TRD) chains contain V, J, and the extra D gene segment, allowing for greater diversity [12] (Figure 1). Augmenting this variability is the process of junctional diversity, where random nucleotide additions or deletions occur at the junctions between gene segments [12]. The numbers of functional genes for every TCR chain are presented in Table 1. Moreover, TCR variable regions contain three complementarity-determining regions (CDR). CDR1 and CDR2 are encoded by the V gene segment and mostly assist in TCR-MHC contact through interactions with the a-helices of MHC proteins [12]. Moreover, CDR3 is encoded by the junctional regions of V-J or D-J gene segments, resulting in hypervariability [12]. This phenomenon is crucial because it is the region that interacts with peptide antigens presented on MHC proteins (forming the pMHC complex) [14].

After a functional TCR is formed, T cells exit the thymus to enter circulation and, consequently, the peripheral naïve T cell compartment [10]. A robust thymus is critical for maintaining TCR repertoire diversity [10]. Peripheral T cell activation by cognate TCR-pMHC signaling and co-stimulatory cues leads to T cell proliferation and clonal expansion, thereby creating a population of T cells with shared TCR sequences during mature T cell mitosis, the T cell clonotype [16]. The resulting TCR repertoire comprises TCRs from antigen-inexperienced naïve T cells as well as from antigen-experienced memory T cells [1]. The TCR repertoire is a dynamic entity, shaped by internal and external noxious stimuli (such as infection, cancer, and autoimmunity) throughout an individual’s life [17]. Exposure to specific antigens triggers the expansion of antigen-specific T cells, leading to changes in the TCR repertoire composition. This process skews T cell diversity toward specific clonotypes—a phenomenon known as TCR bias [18]. These clonotypes are associated with certain conditions and may serve as “molecular barcodes”. Common TCR analysis terms used throughout the text are presented in Table 2.

T cell immune responses are crucial to tackling cancer occurrence, and it is evident that for the purpose of tumor-antigen recognition and generation of a robust immune response, TCR repertoires of high diversity are essential [10].

## 3. TCR Repertoire in MDS

Among all myeloid malignancies, MDS have attracted the most research interest regarding the role of the TCR repertoire for more than two decades. This stems from early evidence of autoimmune processes and T cell dysfunction in MDS pathogenesis, also supported by the observed high response rates to immunosuppressive treatment options, such as anti-thymocyte globulin (ATG) [2,21].

At the beginning of the millennium, Epperson et al. investigated with molecular techniques the TCR profiles of peripheral blood and bone marrow T cells of 15 patients with MDS in comparison with healthy controls and transfusion-dependent non-MDS patients with hemoglobinopathies. The researchers found that patients with MDS demonstrated a significantly higher incidence of skewed TRBV repertoires, with preference to the sub-family TRBV5-3, compared to age-matched controls, with almost identical TCR profiles between peripheral blood and bone marrow T cells, while the families TRBV16, TRBV17, and TRBV23 demonstrated a trend for increased skewing but without reaching statistical significance. Further molecular study of TRBJ usage within the TRBV-skewed families showed a significant TRBJ2-1-specific T cell expansion in MDS patients, providing the first proof of a non-random clonal expansion of T cells in MDS. The same study documented an age-related skewing of TCR repertoires after the age of 60 years in controls, which was not observed in patients with MDS [22]. The age-related skewing of TCR repertoires in the general population was later confirmed, with both cytometric and molecular studies showing a dramatic contraction of the repertoire after the age of 65 years, resulting in the depletion of naïve T cell diversity to less than 1% compared to that before the age of 65 [23].

Clonal T cell expansion based on TCR repertoire restriction was also confirmed in several other studies, along with a higher expression of CD57, a marker of effector CD8+ T cells [24], although some studies found a greater expansion of TRBV families in bone marrow compared to peripheral blood, in contrast to the initial findings [25].

In a later comparative study of TCR repertoires between patients with MDS and controls, Fozza et al. confirmed, using flow cytometry, the expansion of specific TRBV subfamilies in patients compared to the controls, but interestingly, using CDR3 spectratyping, they showed that CD8+ T cell-expanded populations are usually oligoclonal, while CD4+ T cell subsets are mostly polyclonal [26]. Additionally, follow-up of the patients indicated variability in the T cell clonality pattern during the natural course of the disease, with some TRBV subfamilies being preserved and others vanishing after months to years since diagnosis. Given the commonly observed disease progression of MDS to AML, a research group recently compared the TCR repertoire diversity of patients with MDS and AML, and they showed that the peripheral blood CD8+ T cells of patients with MDS exhibit a more confined TCR repertoire diversity compared to that of patients with AML [27].

These findings suggest that in patients with MDS, chronic antigen stimulation likely drives the expansion of CD8+ cytotoxic T cell clones, potentially triggered by aberrant neo-antigen expression or fusion gene products of neoplastic hematopoietic cells in the context of anti-tumor immune surveillance, rather than by viral infections as initially hypothesized. Another possible explanation is that non-randomly expanded CD8+ T cell clones represent an autoimmune attack against bone marrow hematopoietic precursor cells, although both theories remain to be proved [22,26].

Regarding the prognostic role of the abnormal TCR repertoire of peripheral blood and bone marrow in MDS, data have been conflicting, with some researchers demonstrating a correlation between higher IPSS score and increased bone marrow blasts [28], while others reject the association of skewed TRBV families with clinical or laboratory parameters or patient outcomes [25,26]. The reasons for the discrepancy are not clear, although a larger sample size might help elucidate the potential prognostic role of specific TRBV subfamilies.

As it is obvious, research so far has mainly focused on the repertoire of the αβ subtype of the TCR, probably because of the greater abundance of circulating αβ-T cells compared to the γδ-subtype. The latter T cell subpopulation comprises up to 5% of circulating T cells in adults, and most of them usually express the TRDV2 chain [29]. Due to the different TCR architecture, γδ-T cells are capable of recognizing a wide range of both endogenous and exogenous antigens in an MHC-independent manner, exhibiting characteristics of both innate and adaptive immune responses [30]. In patients with MDS, the γδ-TCR repertoire in peripheral blood has also been found profoundly skewed with the use of multi-color flow cytometry. Moreover, these γδ-T cells seem to lack adequate proliferation capacity in response to potent agonists in vitro, an observation that was more evident in patients with concurrent autoimmune disorders [31].

### 3.1. MDS Treatment Effects on TCR Repertoire

Given the established role of T cell dysfunction in MDS pathogenesis, numerous studies have been conducted to assess the effect of different treatment modalities, especially immunotherapy and allo-HSCT, in adaptive immunity, with the rationale that the immunomodulatory effects of different treatments might eventually drive patients’ response. More specifically, several research projects have investigated the possible changes in the TCR repertoire before and after treatment, especially immunotherapy or hypomethylating agents (HMAs), in patients with MDS, as a valuable surrogate of monitoring T cell dynamics in response to treatment.

Multiple immunotherapeutic options have been investigated in patients with MDS, including ICIs (anti-PD-1, anti-PD-L1, anti-CTLA-4, anti-TIM-3), CAR T cells and vaccines, although none of them have been approved by the EMA or FDA so far [5]. Increased TCR diversity has been proposed as a possible prerequisite for successful checkpoint blockade therapy, on the basis that a broader TCR repertoire would increase tumor antigen recognition and, hence, the efficacy of immune surveillance and might result in a favorable outcome with ICIs [10].

#### 3.1.1. Immunotherapy and Hypomethylating Agents

Recently, Lee et al. discovered that among patients with MDS treated with immunotherapy plus HMA responders showed a trend towards increased TCR clonality after treatment, with a significantly higher frequency of novel clonotypes, while non-responders had a contracted TCR repertoire post-treatment. More specifically, anti-CTLA-4 blockade therapy, among the other administered immunotherapies, seemed to induce most changes in clonotypes. Prior to treatment with immunotherapy plus HMAs, responders exhibited significantly lower clonality compared to non-responders, which could therefore be used as a useful predictive biomarker for response to immunotherapy. These findings were interpreted as indicating that the clinical responses of MDS patients to immunotherapy are mainly attributed to the potential anti-tumor activity of novel oligoclonal expanded T cell clones selected from diverse T cell clonotypes, emerging after immunotherapy [32].

The only officially approved pharmacological treatments for higher-risk MDS, HMAs, i.e., azacitidine (5′-AZA) and decitabine, are not typically considered immunotherapies, but they are associated with multiple immunomodulatory effects achieved through regulating gene expression in NK cells, dendritic cells, and T cells [33]. HMA therapy’s effects on TCR repertoire remain inconsistent across studies. The study of Fozza et al. showed that among patients with MDS or AML with myelodysplastic changes, responders to 5′-AZA (only six patients with MDS included) showed a progressive restoration in CDR3 diversity with a decrease in the skewing of TCR repertoire profiles in peripheral blood samples after treatment, especially in the CD4+ T cell subpopulation but with a similar trend in CD8+ T cells, as well [34]. Lee et al. did not observe a significant change in peripheral blood T cell clonotypes in responders to HMA monotherapy [32]. Abbas et al. reported a more profound emergence of novel clonotypes in this subgroup of patients, while non-responders were found to have a contracted bone marrow TCR repertoire after treatment. However, these researchers did not find a significant change in TCR clonality following HMA therapy in either responders or non-responders [35].

Beyond HMAs, in a small study of patients with MDS receiving ATG-based therapy, responders showed regression of dominant T cell clonotypes, while patients without hematological response preserved skewed TRBV repertoires up to 6 months after treatment with ATG [36].

#### 3.1.2. Stem Cell Transplantation in MDS

Regarding allo-HSCT, which is considered the only potentially curative treatment for patients with MDS, little is known so far about its effect on TCR repertoire, with scarce data from patients who have undergone allo-HSCT for indications other than MDS. Within the first year after allo-HSCT, the TCR repertoire diversity changes from a polyclonal profile in donors to a more oligoclonal range in recipients, with TCR repertoire contraction being more evident in the CD8+ subpopulation, while the repertoire diversity remains relatively stable beyond this time-point for the next 5–6 years [37]. The reconstitution of the TCR repertoire of CD8+ T cells in recipients after allo-HSCT seems to be delayed in case T cell depletion with ATG or cyclophosphamide is administered [38]. Finally, immunophenotypic and molecular studies indicate that the recipient’s TCR repertoire is extensively shaped (up to 80%) by the repertoire of memory T cells of the donor pool [38].

In conclusion, with accumulating evidence supporting the dysregulated immune microenvironment and clonal expansion of the T cell compartment in the bone marrow and blood of patients with MDS, there seems to be a need for better understanding the effects of current treatments on T cell clonality and how this could affect patients’ outcomes. Additionally, novel therapies that could potentially restore T cell diversity and improve patients’ anti-tumor immune surveillance might be the solution to the suboptimal response of some patients to existing treatments. Interestingly, apart from prognostic and predictive biomarkers, TCR repertoire metrics might also provide useful information about the toxicity profile of immunotherapy based on a specific patient’s TCR repertoire [12].

## 4. TCR Repertoire in AML

The heterogeneity of AML is reflected in the diverse T cell immune responses [39]. The dysfunctionality of the T cell compartment is driven by multiple interacting factors that are either AML-intrinsic or in the leukemic microenvironment [2]. The intrinsic immune evasion properties of AML blast include immunoediting properties that lead to downregulation of MHC class I/II molecules [40,41]. The propagation of ineffective T cell states (like senescence and exhaustion) [2,42], reduced ability to form an immune synapse [43], and increased numbers of regulatory T cells (Tregs) [2] are some of the features of the T cells in AML. On the other hand, the anti-leukemic activity of T cells is evidenced by the Graft-versus-Leukemia (GvL) of allo-HSCT, along with the observations that increased BM T cells and increased T cells post-chemo are associated with better overall survival (OS) in AML patients [44,45].

The TCR repertoire and corresponding clonal dynamics of the T cell space in AML reveal another level of interactions between AML and the immune system in different disease states and therapeutic interventions. Intuitively, in the context of AML, the T cells are expected to show clonality, with the corresponding TCR repertoire being skewed as a response to leukemia-associated antigens and neoantigens. Early studies on acute monoblastic leukemia, which analyzed the TRBV subfamilies associated with CDR3 by PCR and GeneScan, revealed the oligoclonal expansion of T cells in many TRBV subfamilies, with the TRBV2 subfamily being of notice [46]. Similarly, in the case of acute promyelocytic leukemia, TRBV skewing was evident for the TRBV10, TRBV23, TRBV3, and TRBV21 subfamilies in most of the patients [47]. Ou Y et al. designed a novel panel of PCR primers for the entirety of all functional TRBV genes and not only CDR3 sequences, based on the international immunogenetics (IMGT) database [48]. All the AML patients studied showed mono- or oligoclonal expansion of the TRBV15 subfamily. Additionally, clonal expansions of TRBV2, TRBV4, TRBV6, and TRBV13 were identified in some patients [48].

Regarding the role of γδ T cells, TRDV subfamilies show significant restriction in AML patients, with TRDV4 and TRDV8 being the most frequently expanded clonotypes [49]. TRDV4 and TRDV8 might potentiate complete remission (CR) in these patients, whereas TRDV5 and TRDV6 expression was more frequent in the disease relapse [49]. TRDV restriction (especially of TRDV8), along with lower TRDV4 frequencies, were also observed in a subset MDS of patients with refractory anemia with excess blasts (RAEBs) that ultimately progressed to AML [50]. A TRGV analysis did not produce a substantial signature in these patients [50]. On the opposite side, Kong X et al. reported an oligoclonal TRGV repertoire with diminished TRGV gene expression levels compared to the controls [51]. Remarkably, increased TRGV9 expression and increased circulating Vγ9+Vδ2+ T cells correlated with superior prognosis [51]. Additionally, appropriately stimulated Vγ9+Vδ2+ T cells could recognize and eradicate AML cells in vitro, while Vγ9+Vδ2+ T cell-treated mice had superior survival in a mouse disease model [52]. Vδ1+ T cells also showed promise as candidates for adoptive cell immunotherapy [53].

A novel in silico analysis by Zhang et al. of pediatric and adult AML “bulk” TCR seq data revealed a restricted TCR repertoire in all age groups compared to the control [19]. Of note, infant AML samples showed the least T cell clonal expansion among age groups, and consistently, the most virus-specific TRB-CDR3s were observed in the adult sub-group. An investigation of the mutations and gene fusions on αβ T cell activation unclothed the immunogenic potential of *CBFB-MYH11* fusions, especially in the pediatric population [19]. Opposingly to normal controls, the γδ T cell space showed an age-associated increase. Moreover, by clustering TRD-CDR3 sequences, the authors revealed in some patients a conserved pattern of TRD-CDR3s associated with TRDV2 and TRDJ3, with a positive impact on prognosis [19]. A consequent study utilized two different computational tools to investigate the TRA and TRB profiles with clinical and molecular characteristics from AML patients. A skewed TCR profile response is related to a higher white blood cell (WBC) as well as BM and peripheral blood (PB) blast percentages, with only *WT-1* mutations (among other genetic lesions) being negatively correlated with TRA and TRB unique clone counts after multivariate regression [54]. Moreover, Treg marker foxp3, cytotoxic marker Granzyme B provide (GMZB), and exhaustion markers were positively correlated with TRA and TRB unique clone counts [54]. Coupled methylomics and TCR analysis studies also provide additional knowledge on the dynamics of the TCR repertoire. While methylation patterns across TCR loci may be conserved among AML patients, they are characterized by variable DNA methylation of the TRAV and TRBV gene loci, affecting the interindividual variability in the TCR repertoire [55]. Many TRAV and TRBV loci are found to be differentially methylated in the T cells from AML patients. Of note, TRAV9-1 and TRBV5-1.2 showed the lowest methylation levels in T cells from AML patients, whereas TRAV8-4.2 and TRBV4-2.1 showed the highest methylation levels [55].

In vitro experiments of TCR responses in different experimental settings showed the variability of clonal dynamics coupled with functionality and phenotypic changes. In vitro T cell stimulation by patient-derived leukemic-dendritic cells (DC/DCleu) and blast cells produced significant TRBV and TRBJ restriction, particularly in CD8+ T cells [56]. Notably enough, in two cases with balanced CD4:CD8 ratios, anergy and blast growth were observed after T cell stimulation [56]. Cytokine stimulation may partially invigorate T cell responses, as shown by TCRζ upregulation with concomitant TRVB polyclonal expansion, although the effects may be variable and contradicting due to disease post-chemotherapy [57].

### 4.1. AML Treatment Effects on TCR Repertoire

An investigation of the clonotypic diversity and evolution under the pressure of chemotherapy and immunotherapy is important to understand the immunologic factors of treatment failure and disease progression. Feng Z et al. analyzed the TRB CDR3s of CD8+ T cells through deep sequencing in a heterogeneous group of AML patients pre- and post-chemotherapy (daunorubicin and cytarabine) [58]. A skewed TCR repertoire with clonal expansion (especially in the BM) of AML patients was observed, with higher ratios of identical T cell clones in the AML BM [58]. Significant clonal expansion with reduced diversity was observed in AML patients who relapsed after chemotherapy compared to CR patients, with TRBV gene segment usage changing drastically after relapse [58]. Moreover, reduced TRB diversity was associated with PD-1 positivity in CD8+ T cells [58].

#### 4.1.1. Hypomethylating Agents

HMAs, used in the treatment of AML, possess immunomodulating properties with effects across TCR gene loci through epigenetic modifications. A combination of 5′-AZA and chemotherapy did not change the T cell diversity indices in a diverse group of AML patients, although better event-free survival (EFS) and overall survival (OS) were associated with a higher pretreatment TRB diversity and consequent rise in T cell richness after 5′-AZA administration, along with significant TRBV-J skewing [59]. Moreover, T cell clustering by shared antigen specificity revealed shared clusters between the controls and post-treatment responders, which might imply partial repertoire skewness [59]. Differential TRBV usage was also associated with response to 5′-AZA in the study by Beckford et al. [60]. On the contrary, 5′-AZA treatment demonstrated improvement in CDR3 diversity with a reduction in TRBV skewness, in a cohort of patients with multilineage dysplasia AML [34]. Pospiech et al. showed that treatment with HMAs also produced a decrease in overall methylation values in skewness T cells; however, no significance was observed in the methylation of individual TRAV and TRBV gene loci [55]. Even though the interaction between HMAs and the TCR repertoire changes merit further study, the studies reveal useful insights about the potential role of the TCR as a response marker.

#### 4.1.2. Immune Checkpoint Inhibitors

Further interrogation of the CD8+ T cell subset is crucial for the delineation of anti-leukemic responses and the advancement of immunotherapy in AML. Through paired single cell (sc) RNA-sequencing (seq) and scTCR-seq in newly diagnosed and relapsed/refractory (R/R) AML samples—followed by a battery of bioinformatics methods—profiling of the CD8+ T cells from R/R patients showed clonal hyperexpansion (especially in the cytotoxic effector group) with a terminal differentiation state and gene signature, in contrast to a more plastic T cell state in newly diagnosed patients, owing to chronic antigen stimulation [61]. Most clonotypes were patient-specific (termed unknown specificity), with few shared clonotypes being pathogen-related [61]. ICI-based immunotherapy produced expansion of cytotoxic T cell clonotypes, particularly in patients with a response or stable disease after therapy, with TCR repertoire contraction in therapy-resistant patients [62]. The stem- and memory-like T cell subpopulation [(CD8+ Granzyme K (GZMK)+] phenotype was the most enriched in responders, which interestingly showed shared clonotypes with cytotoxic CD8+ T cells post-treatment [62]. Thymic dysfunction and involution immediately affect TCR repertoire diversity and consequently immunotherapy success, with many pre-clinical and clinical rejuvenation strategies in development (reviewed in [63]).

#### 4.1.3. Stem Cell Transplantation in AML

Undoubtedly, TCR repertoire changes in the context of SCT are the most studied among available treatment modalities. Many studies have documented the development of a skewed TCR repertoire following a transplant procedure, although conflicting results apropos to the correlation of repertoire diversity and clinical outcomes exist. An early study demonstrated TRAV and TRBV skewing in the early post-transplant period, especially in allo-HSCT recipients, in a heterogeneous group of patients [64]. Another study evinced that similar patterns of TRBV skewing post-allo-HSCT can also be recapitulated in vitro [65]. Yew P. et al. investigated TCR repertoire changes in allo-HSCT recipients from matched and haplo-cord donors through NGS methods [66]. Allo-HSCT was associated with a significant reduction in TRA and TRB diversity in the post-transplant setting, with a subsequent rise in TCR diversity correlating with higher cord-chimerism during the early post-transplant period [66]. Residual thymic function is substantial regarding the rate of T cell reconstitution and generation of repertoire diversity following allo-HSCT [67]. Patients affected by Graft-versus-Host Disease (GVHD) showed substantial clonal expansion of specific T cell clonotypes, leading to reduced TCR diversity—particularly among those without disease relapse. Conversely, the non-GVHD/non-relapsed subgroup of patients exhibited higher TCR diversity [66]. The authors also reported the expansion of similar TRAV-J (TRAV38-2/DV8-TRAJ30) and TRBV-J (TRBV15-1/TRBJ2-1) combinations associated with shared HLA alleles, albeit only in two GVHD patients [66]. Further elucidation of those differential TCR clone changes is essential to setting apart GVHD from GVL effectors. In a cohort of AML patients in remission, TRB sequencing demonstrated that post-allo-HSCT, there is an increase in T cell clonality with clonotype expansion; however, no overlap of clonotypes between samples was shown, probably indicating the antigenic variety of GVL [68]. Arguably, a microtransplantation-based regiment in elderly AML patients produced a persistent polyclonal expansion pattern [69]. In relapses after allo-HSCT—especially in HLA-matched procedures—exhausted stem/memory-like and central memory T cells are increased, with an escorting restricted TCR repertoire that is leukemia-reactive, although with impaired effector capacities [70]. These findings are interesting in light of the aforementioned studies by Abbas H. et al. and Desai P. et al. [61,62], which warrant further investigation.

Deep TRB sequencing of T cells from donor lymphocyte infusion (DLI) recipients revealed that CD8+ T cells (but not CD4+) had diminished TRB diversity with collateral clonal expansion upon GVL establishment [71]. Of particular interest is the fact that expanded preexisting and newly developed CD8+ T cell clones, in recipients with GVL, demonstrated significant overlap at different time points [71]. Another post-DLI TRB analysis showed that GVHD with an established GVL effect is linked to a more diverse response, skewed to a broader range of minor histocompatibility antigens (MiHAs), whereas patients with only GVL had a drop in TCR diversity [72].

The use of autologous stem cell transplantation (auto-SCT) in AML has been revisited as a viable option in selected patient populations. Recent studies [73] highlight its role in inducing remission in patients with favorable prognostic markers. Post-auto-SCT, the TCR repertoire shows a reconstitution of clonal diversity, particularly in cytotoxic and memory T cells. This suggests a potential for immune-mediated control of residual disease. Studies have shown that some memory and cytotoxic BM T cells survive ex vivo pharmacologic eradication and assist in post-SCT immune reconstitution in pediatric AML [74,75]. Besides the demonstration of T cell clonotypic perseverance before and after auto-SCT, the authors reported a polyclonal T cell population prior to transplantation, probably due to the fact that the studied patients were in CR [74,75].

Post-transplantation cytomegalovirus (CMV) infection is a frequent and serious complication that immediately affects the TCR repertoire. Studies have shown persisting TRA and TRB repertoire restriction, with CMV-specific CD8+ clonotype expansion [76,77], with one study reporting the drop in effector memory CD8+ T cell diversity [76]. The influence of this specific clonotypic expansion at the expense of a greater anti-leukemic and anti-microbial diversity on allo-HSCT outcomes needs further analysis.

Concerning the role of γδ T cells in the post-SCT setting, there is an association of increased γδ T cell numbers and improved outcome [78,79]. In an AML patient population, Arruda L. et al. analyzed the influence of TRG diversity with donor CMV positivity and outcome after peripheral blood stem cell (PBSC) transplantation [80]. Overall PBSC grafts demonstrated a diminished TRG diversity without any association with relapse or acute GVHD. However, patients who did not relapse showed a higher degree of clonal expansion and more public clonotypes, with different V-J segment expression and combinations (albeit TRGV9 and TRGJP were the most frequently expressed segments in all patients) [80]. CMV positivity led to dominance of the hyperexpanded clones with less public clonotypes, although a singular CD3 TCR sequence (CATWDGPYYKKLF) was shared among all patients [80].

To summarize, evidence suggests that the T cell response in AML is characterized by diminished TCR variety, with fluctuations apropos to disease stage and outcomes. However, further larger studies must be conducted, considering the great disease heterogeneity of AML and specific T cell phenotypes, to discover specific immune responses and even leukemia-associated antigens. This will aid in the development of more effective immunotherapies.

**Figure 2:**
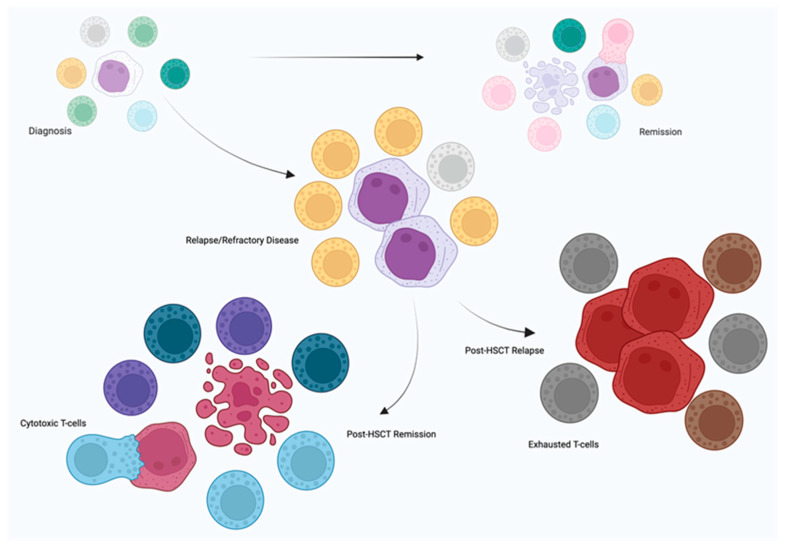
Representation of TCR repertoire dynamics in the natural history of MDS and AML. A skewed TCR response in diagnosis is followed either by increased diversity and novel clonotypes (during therapy response) or further reduction in TCR diversity and clonal hyperexpansion (in relapsed/refractory disease). Allo-HSCT effects on TCR repertoire are variable and interrelated with outcome. Different colored T cells represent different clonotypes, and different colored myeloid cancer cells represent phenotypes of disease stages. Created in BioRender. Barakos, G., https://BioRender.com/l59m745 (accessed on 3 December 2024).

The complexity of the T-clonotype responses in MDS and AML, as well as a comparison of the TCR responses between the two entities, are presented in Figure 2 and Table 3, respectively. Studies investigating a correlation between TCR alterations and outcome are presented in Table 4.

## 5. Future Directions

To advance our understanding of the TCR repertoire dynamics in MDS and AML, several key research priorities emerge. High-resolution single-cell multi-omics approaches combining TCR sequencing with transcriptomics and epigenetics will be crucial for understanding the relationship between TCR profiles and T cell states. Longitudinal studies tracking TCR repertoire changes from diagnosis through treatment and remission/relapse will help identify predictive patterns. The standardization of TCR analysis methods and metrics across studies remains essential for meaningful cross-study comparisons and validation of TCR repertoire metrics as clinical biomarkers.

**Figure 3:**
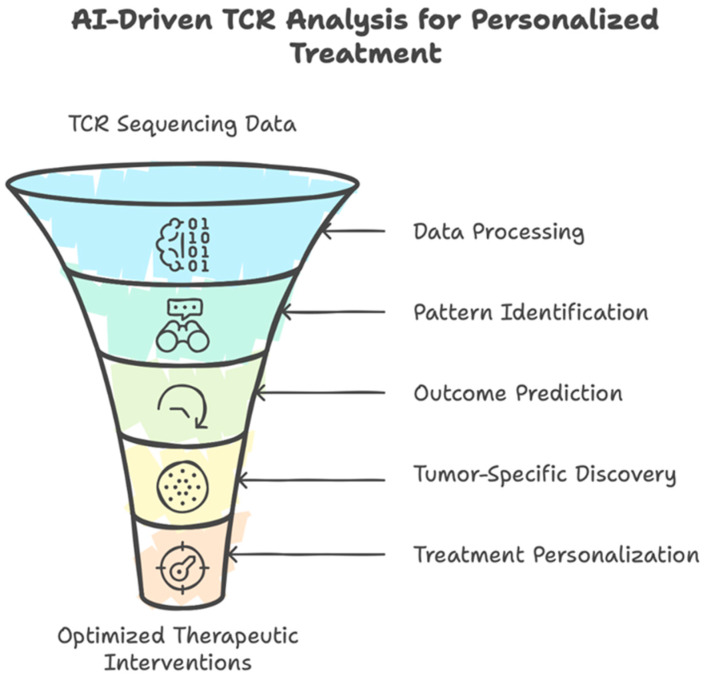
The integration of artificial intelligence (AI) and machine learning (ML) may transform the TCR repertoire analysis and contribute to personalized treatment.

Among these priorities, the integration of artificial intelligence (AI) and machine learning (ML) tools into TCR repertoire analysis presents perhaps the transformative opportunity to decode the immense complexity of immune responses in MDS and AML [81]. AI-driven algorithms, particularly those leveraging deep learning and neural networks, can process high-dimensional sequencing data, identifying subtle patterns and correlations that are often imperceptible to traditional analysis methods. Recent advances in machine learning have revolutionized TCR binding prediction, enabling more accurate identification of antigen-specific responses [82]. By combining AI-based insights with experimental validation, researchers can streamline biomarker discovery, improve risk stratification, and personalize treatment plans (Figure 3). These computational approaches are particularly valuable for 1. pattern recognition and biomarker discovery, e.g., identification of TCR signatures associated with disease progression or prediction of treatment responses based on TCR repertoire characteristics; 2. therapeutic applications, e.g., design of more effective CAR-T cell therapies through optimal TCR selection or development of personalized neoantigen vaccines; 3. clinical decision support, e.g., risk stratification based on TCR repertoire patterns; and 4. data integration and analysis, e.g., integration of multi-modal data (genomic, transcriptomic, and TCR sequences). Current examples of ML applications in cancer immunology include, among others, GENTLE (Generator of T cell receptor repertoire features for machine learning), a web-based tool for feature selection of cancer-related TCR data [83]; BertTCR, a deep learning model trained in predicting cancer-related TCR responses [84]; and iCanTCR, a deep learning framework utilized in TCR-based early cancer detection [85]. Recent work has demonstrated that machine learning models can achieve unprecedented accuracy in predicting TCR–peptide binding, potentially revolutionizing our ability to develop targeted immunotherapies [82]. Furthermore, AI-based approaches have shown promise in predicting treatment outcomes by associating specific TCR repertoire metrics with clinical responses to immunotherapy or chemotherapy [81].

Beyond computational advances, investigating the interplay between the TCR repertoire and other immune components like B cells, NK cells, and myeloid cells will provide a more complete picture of the immune landscape in these diseases.

The continued development of AI/ML tools specifically designed for TCR analysis, combined with standardized analysis methods and comprehensive clinical validation studies, promises to accelerate our understanding of immune responses in MDS and AML. This integrated approach, bringing together advanced sequencing technologies, sophisticated computational methods, and clinical insights, will be essential for translating the TCR repertoire analysis into practical clinical applications that improve patient care through more personalized treatment approaches.

## 6. Limitations

Several significant limitations must be acknowledged when analyzing the existing literature on TCR repertoire in MDS and AML. Many studies had relatively small sample sizes, which constrain their statistical power and the generalizability of their results. The variability in patient demographics, disease stages, and treatment regimens among research complicates direct comparisons. The absence of consistency in TCR analysis methodologies and metrics among various research groups hinders meta-analyses and the determination of clinically significant thresholds.

Current TCR sequencing methodologies are likewise constrained by technical restrictions. Bulk sequencing techniques may overlook infrequent clonotypes and are unable to accurately associate TCR sequences with particular T cell phenotypes or functional states. Moreover, the majority of research concentrates on certain time intervals instead of offering longitudinal data across illness development and treatment. This constrains our comprehension of TCR repertoire dynamics and their correlation with clinical outcomes. The limited amount of research analyzing both αβ and γδ TCR repertoires concurrently creates deficiencies in our understanding of their coordinated responses. Although connections between TCR measures and clinical outcomes have been shown, their causative links and underlying processes frequently remain ambiguous, underscoring the necessity for more mechanistic investigations.

## 7. Conclusions

The exploration of the TCR repertoire in MDS and AML has opened a new frontier in understanding the immune landscape of these malignancies. The great complexity of the immune milieu of MDS and AML is reflected in the modest success of novel immunotherapies. Our review only illuminates some aspects of the TCR repertoire dynamics of these myeloid malignancies. TCR skewing in different disease phases suggests the presence of elusive antigens. We urgently need well-designed, prospective clinical studies to evaluate the dynamic changes in TCR repertoires at multiple disease stages and treatment time points, as well as standardization of the most appropriate molecular or immunophenotypical techniques for TCR analysis for more robust evidence. By integrating advanced TCR sequencing technologies with clinical and molecular data, we can identify novel biomarkers for diagnosis, prognosis, and therapeutic response. Collaborative efforts between immunologists, oncologists, and bioinformaticians will be essential to harness the full potential of the TCR repertoire analysis, paving the way for personalized immunotherapeutic strategies.

## Figures and Tables

**Table 1:** Numbers of functional gene segments of TCR chains. TCR: T cell receptor, TRA: T cell receptor alpha, TRB: T cell receptor beta, TRG: T cell receptor gamma, TRD: T cell receptor delta, V: variable, D: diversity, J: joining, C: constant [15].

TCR Chain	V Segments	D Segments	J Segments	C Region
TRA	44–46 (33–35 subgroups)	-	50	1
TRB	40–48 (21–23 subgroups)	2	12–13	2
TRG	4–6	-	5	2
TRD	7–8 (7–8 subgroups)	3	4	1

**Table 2:** Commonly used metrics in TCR repertoire analysis (table not exhaustive) [12,19,20].

Metric	Definition
Density	Proportion of T cells within a specific area
Richness	Number of unique TCR sequences in a sample
Evenness	Distribution spectrum of TCR sequences
Diversity	Richness and evenness of clonotypes in a sample
Clonality	Proliferation of specific clonotypes within a sample
Shannon entropy	A measure of TCR diversity that accounts for both the richness and evenness of clonotypes within a sample (higher values indicate greater diversity)
(Gini-) Simpson index	A measure of TCR diversity reflecting the probability that two randomly selected clonotypes from a sample are different (higher values represent greater diversity)
Jaccard index	TCR overlap between samples
Morisita index	Frequency of shared TCRs between samples
Public clonotype	Clonotypes shared among samples
Private clonotype	Clonotypes unique to specific samples
CPK (CDR3s per kilo of TCR reads)	An estimation of TCR diversity in “bulk” NGS data (higher values represent greater diversity)
Normalized unique clone count	An estimation of TCR diversity in “bulk” NGS data (higher values represent greater diversity)

**Table 3:** Side-by-side comparison of the TCR repertoire in MDS and AML.

	MDS	AML
Clonality	Oligoclonal expansion of CD8+ T cells Polyclonal expansion of CD4+ T cells Absence of age-related skewness in MDS patients More confined CD8+ TCR repertoire, in the peripheral blood, compared to AML	Oligoclonal (or even monoclonal) T cell expansions Substantially skewed TCR at diagnosis and relapse/refractory disease
TCR gene usage	Skewed TRBV5-3 (TRBV16, TRBV17, TRBV23 showing trends of skewing) Clonal expansion of TRBJ2-1	Oligoclonal expansion of TRBV15, TRBV2 (especially in acute monoblastic leukemia), TRBV4, TRBV6, TRBV13 Skewed expression of TRBV10, TRBV23, TRBV3, TRBV21 in acute promyelocytic leukemia
γδ T cells	Skewed γδ TCR repertoire, with inadequate proliferation capacities	γδ T cells demonstrate a restricted TRDV repertoire with specific clonotypes (TRDV4, TRDV8) linked to favorable outcomes and others (TRDV5, TRDV6) associated with relapse Subset of MDS patients (e.g., RAEB progressing to AML) also shows TRDV8 restriction and lower TRDV4 frequencies Oligoclonal TRGV repertoire with diminished TRGV gene expression in AML patients
Effects of HMAs	Inconsistent results Some studies show restoration of TCR diversity and novel clonotype emergence	Potential improvements in diversity and skewness reduction linked to better outcomes. Pre-treatment TCR diversity and post-treatment repertoire changes might serve as useful biomarkers for therapy response Decrease in methylation values in skewed T cells but without locus-specific significance
Effects of Immunotherapy	Novel clonotype emergence and increased clonality, especially in responders ATG induces regression of dominant clones in responders	Expansion of effective cytotoxic and memory-like T cell clonotypes in responders Resistance to therapy correlates with TCR repertoire contraction and lack of clonotype adaptation
Effects of SCT	Clonality transition from a polyclonal (reflecting a diverse donor pool) to a more oligoclonal repertoire in the first year post-transplant, especially in the CD8+ compartment	Significant TRAV and TRBV skewing occurs early after allo-HSCT SCT induces significant changes in the TCR repertoire, with diversity, clonality, and clonotype expansions fluctuating depending on GVHD, GVL, relapse, and CMV infection

**Table 4:** Summarized evidence of TCR/clonotype changes with possible outcome implications. Only studies that explicitly investigated alterations in TCR metrics and different outcomes are included.

Reference	Population	Methods	Therapy	TCR/Clonotype Change	Prognostic/Predictive Correlations
Jin et al. [49]	Newly diagnosed AML patients	RT-PCR, GeneScan	N/A	TRDV4, TRDV8 oligoclonality	Protective factor for CR
				TRDV5, TRDV6 oligoclonality	Relapse
Geng et al. [50]	RAEB MDS patients	RT-PCR, GeneScan	N/A	Reduced TRDV4 frequencies	Progression to AML
				TRDV8 clonal expansion	
Kong et al. [51]	Newly diagnosed de novo AML patients	RT-qPCR, GeneScan	Various Chemotherapy regimens HSCT	Higher TRGV expression levels	Improved OS
				Increased TRGV9 usage Increased circulating Vγ9+Vδ2+ T cells	Protective factor for CR
Zhang et al. [19]	TCGA and TARGET AML data	In silico, NGS	Various Chemotherapy regimens	Specific TRD-CDR3 cluster associated with TRDV2, TRDJ3 usage	Superior OS
Pospiech et al. [55]	TCGA AML data	In silico, NGS	Various Chemotherapy regimens	Normalized unique TRA/TRB counts	No association with survival
				Public clonotypes (CANTGELFF TRB CDR3 clone)	Superior OS
Feng et al. [58]	Newly diagnosed AML patients	NGS	DNR + AraC	CD8+ TRB clonotype expansion Decreased Shannon entropy Increased TRB clonotype evenness	Disease relapse
Grimm et al. [59]	De novo and secondary AML patients	NGS	5′-AZA +/− Mitoxantrone + AraC	Higher pre-treatment TRB diversity (increased Shannon entropy) Increase in post-treatment TRB richness TRBV skewing (TRBV12-3, 5-7, 6-9)	Superior EFS and OS
Beckford et al. [60]	Newly diagnosed AML patients	NGS	5′-AZA	TRBV16, TRBV12-5 increase post-therapy TRBV12-2 increased pre-therapy	Response to 5′-AZA
				TRBV7-4 increase post-therapy	No response to 5′-AZA
Abbas et al. [62]	R/R AML patients	NGS	5′-AZA + Nivolumab	αβ T-clonotype expansion	Response to therapy/stable disease
				αβ T-clonotype contraction	Resistance to therapy
Yew et al. [66]	AML (and 1 MDS) patients	NGS	allo-HSCT (MD+ Haplo-cord)	Early increase in TRA/TRB diversity	Improved cord chimerism
				Increased TCR diversity	Remission and no GVHD occurrence
				Decreased TCR diversity	Remission and GVHD occurrence
Schultze-Florey et al. [71]	AML patients with relapse or increased host chimerism after allo-HSCT	NGS	DLI	Decreased CD8+ TRB diversity Clonotype expansion With pre- and post-treatment overlap	GVL occurrence and durable remmission
				Absence of clonotype expansion	Relapse
van Bergen et al. [72]	AML, CML, MDS patients with relapse after allo-HSCT	NGS	DLI	Reduced TRB diversity of MiHA-reactive CD8+ clones	GVL with CR and no GVHD emergence
				Increased TRB diversity to broader MiHAs	GVL with concomitant GVHD occurrence
Arruda et al. [80]	AML patients	NGS	PBSC	TRG clonotype expansion Lower usage of TRGV4-J2, TRGV5-J2, TRGV8-JP2 Higher usage of TRGV2-JP1, TRGV9-JP Increased public clonotypes	Non-relapse
				Overall TRG status	Not associated with acute GVHD development
				Lower TCR diversity More private clonotypes	Donor CMV positivity
Lee et al. [32]	MDS and CMML patients	FC, PCR, in silico	IT + HMAs	Emergence of novel clonotypes vs. TCRB repertoire contraction	Responders vs. non-responders
			HMAs	No change in TCR clonality	None
Fozza et al. [34]	MDS and AML	FC, PCR	5′-AZA	Restoration of CDR3 diversity	Response to 5′ AZA
Abbas et al. [35]	MDS	PCR, in silico	HMAs	Emergence of novel clonotypes vs. TRB repertoire contraction	Responders vs. non-responders
Kochenderfer et al. [36]	MDS	FC, PCR	ATG-based treatment	Regression of dominant TCR clonotypes	Response to ATG

Abbreviations: 5′-AZA: azacitidine, AraC: cytarabine, ATG: antithymocyte globulin, CDR3: complementarity determining region 3, CMV: cytomegalovirus, CR: complete remission, DLI: donor lymphocyte infusions, DNR: daunorubicin, EFS: event-free survival, FC: flow cytometry, GVHD: Graft-versus-Host disease, GVL: Graft-versus-Leukemia, HMAs: hypomethylating agents, MD: matched donor, MiHA: minor histocompatibility antigens, NGS: next-generation sequencing, OS: overall survival, PBSC: peripheral blood stem cell transplantation, RT-PCR: real-time polymerase chain reaction, TARGET: therapeutically applicable research to generate effective treatments, TCGA: Tumor Cancer Genome Atlas, TRA: T cell receptor alpha, TRB: T cell receptor beta, TRG: T cell receptor gamma, TRD: T cell receptor delta.
